# Far East Scarlet-Like Fever Caused by a Few Related Genotypes of *Yersinia pseudotuberculosis*, Russia

**DOI:** 10.3201/eid2203.150552

**Published:** 2016-03

**Authors:** Nelly F. Timchenko, Ruslan R. Adgamov, Alexander F. Popov, Ekaterina K. Psareva, Konstantin A. Sobyanin, Alexander L. Gintsburg, Svetlana A. Ermolaeva

**Affiliations:** Institute of Epidemiology and Microbiology named after G.P. Somov, Vladivostok, Russia (N.F. Timchenko, E.K. Psareva);; Gamaleya Institute of Epidemiology and Microbiology, Moscow, Russia (R.R. Adgamov, K.A. Sobyanin, A.L. Gintsburg, S.A. Ermolaeva);; Paсific State Medical University, Vladivostok (A.F. Popov);; Pirogov Russian National Research Medical University, Moscow (S.A. Ermolaeva)

**Keywords:** *Yersinia pseudotuberculosis* infections, multilocus sequence analysis, clonal evolution, bacterial CNF1, yopE protein (Yersinia), bacteria, Enterobacteriaceae, bacterial infections, scarlet fever, skin diseases, Russia, Far East scarlet-like fever, FESLF

## Abstract

We used multivirulence locus sequence typing to analyze 68 *Yersinia pseudotuberculosis* isolated in Russia during 1973–2014, including 41 isolates from patients with Far East scarlet-like fever. Four genotypes were found responsible, with 1 being especially prevalent. Evolutionary analysis suggests that epidemiologic advantages could cause this genotype’s dominance.

Far East scarlet-like fever (FESLF), a rare and poorly studied disease caused by *Yersinia pseudotuberculosis*, was first described in 1959, when an outbreak involving >300 hospitalized patients occurred in the city of Vladivostok, Russia, on the coast of the Pacific Ocean (*1*). Since the 1960s, multiple outbreaks and sporadic cases of FESLF, mainly associated with consumption of contaminated vegetables, have been reported from far eastern and northern parts of Russia and other countries in Eurasia (*2**–**4*).

Comparing clinical patterns of FESLF and pseudotuberculosis showed that FESLF is not just a form of pseudotuberculosis but is an independent infectious disease that was unknown until the 1960s (*4*). FESLF is an acute disease with a cyclic course that includes severe fever and early signs such as rash that covers the body, particularly the face, neck, toes, and hands; these signs have become known as “hood,” “gloves,” and “socks” (Technical Appendix Figure 1). Typical features of FESLF include a “raspberry tongue” and well-defined nipples. Erythema nodosum can occur with relapse; lamellar or defurfuration on earlobes, hands, palms, feet, and trunk appears during the recovery period. We sought to determine clonal relationships of *Y. pseudotuberculosis* strains responsible for cases of FESLF reported in Russia during 1973–2014 and environmental strains found in vegetables and small rodents.

## The Study

**Figure 2:**
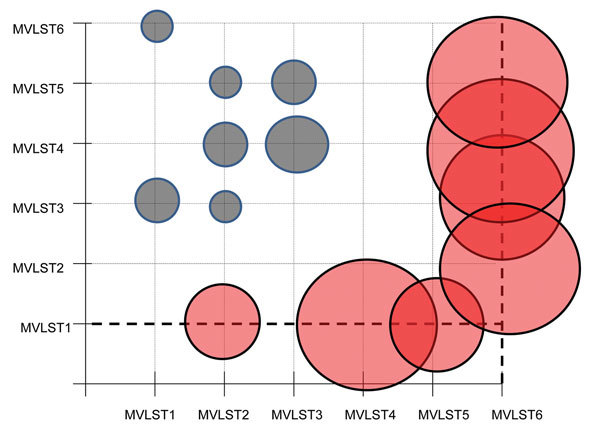
Graphic representation of the evolutionary analysis that tested the hypothesis of equality of evolutionary rates between multivirulence locus sequence type (MVLST) genotypes for study of Far East scarlet-like fever caused by a clonal group of *Yersinia pseudotuberculosis*, Russia. The χ^2^ test statistic was applied for the pairwise comparison of concatenated sequences of MVLST markers, with the *Y. pestis* sequence being used as an outgroup. Circles indicate values of the χ^2^ test statistic of the pairwise comparison calculated in MEGA6 (*10*); diameters correspond to values of rejection of the null hypothesis that states the equality of evolutionary rates between pairs of concatenated sequences. Statistically significant values are shown in red.

Our study examined 68 isolates collected in Russia during 1973–2014, including 17 outbreak and 24 sporadic isolates from humans and 15 rodent and 11 vegetable isolates (Technical Appendix Table 1, Figure 2). All but 3 isolates belonged to the O1b serotype; these 3 isolates belonged to the O3 serotype. The most recent FESLF isolates (from 2014) came from a patient who showed typical signs of FESLF, including a cyclic course, fever, and “raspberry tongue.” A comparison of clinical signs and symptoms in historical versus recent patients suggested that the disease had not evolved since its first description.

The isolates were kept frozen until the experiment started. To characterize clonal relationships of the strains, we applied the multilocus sequence typing (MLST) scheme developed by Laukkanen-Ninios et al. (*5*). PCR products were obtained with primers and conditions listed at the *Y*ersinia pseudotuberculosis MLST database (University of Warwick, Coventry, UK; http://mlst.ucc.ie/mlst/dbs/Ypseudotuberculosis).

We found 3 MLSTs among FESLF isolates: MLST2 (n = 33), MLST26 (n = 5), and MLST32 (n = 3); this MLST was specific for serotype O3 (Table 1). All but 1 vegetable isolate belonged to MLST2, which was also found in 9 (60%) of 15 rodent isolates. MLST2 prevailed among isolates from all sources.

**Table 1:** Combined genotypes of the *Yersinia pseudotuberculosis* strains in study of Far East scarlet-like fever caused by a clonal group of *Y. pseudotuberculosis*, Russia*

MVLST	Source of isolated strains, no.			MLST†	VST‡	Plasmid profiles		
	FESLF	Rodents	Vegetables			pYV§	pYpsIP31758.1¶	pVM4.4#
1**								
** A**	29	9	10	2	1	+	+	–
** B**	4	0	0	2	1	+	–	+
**2**	5	0	0	26	1	+	–	–
**3**	3	1	0	32	2	+	–	–
4	0	0	1	14	3	+	–	–
5	0	3	0	42	4	+	–	–
6	0	2	0	64	2	+	–	–

*Multivirulence locus sequence typing (MVLST) types found in Far East scarlet–like fever (FESLF) isolates are in bold. MLST, multilocus sequence typing; *VST*, virulence sequence types; +, positive; –, negative. †MLST types are provided in the *Y. pseudotuberculosis* MLST database (http://mlst.ucc.ie/mlst/dbs/Ypseudotuberculosis). ‡VSTs are determined on the basis of alleles of the virulence genes *inv*, *cnf*, *yadA*, and *yopE*. §Plasmid designated pYV was evidenced by PCR with primers specific to *yadA* and *yopE* (Technical Appendix Table 2, http://wwwnc.cdc.gov/EID/article/22/3/15-0552-Techapp1.pdf) and confirmed by agarose gel electrophoresis. ¶Plasmid designated pYpsIP31758.1, which is also called pVM82 (*3*), was confirmed by PCR with primers specific to *dotA* (Technical Appendix Table 2) and was confirmed by agarose gel electrophoresis. #Plasmid designated pVM4.4 was confirmed with agarose gel electrophoresis. **The subtypes had different plasmid profiles.

MLST analysis was complemented with sequencing of 4 virulence genes involved in critical steps of generalized infection: intestine barrier crossing (*inv* and *yadA*) (*6**,**7*) and macrophage activity regulation (*yopE* and *cnf*) *(**8**,**9*) (Tables 1, 2). The genes *inv* and *cnf* are chromosomal, whereas *yopE* and *yadA* are encoded on the virulence plasmid of Yersinia (pYV). Sequences from this study have been deposited into GenBank (accession nos. KR028003–KR028011). A total of 4 distinct virulence sequence types (VSTs) were found (Table 1).

**Table 2:** Polymorphism of housekeeping and virulence genes in study of Far East scarlet-like fever caused by a clonal group of *Yersinia pseudotuberculosis*, Russia*

Target gene†	Fragment length, bp‡	Alleles, no.	Indels, no.	Polymorphic sites/parsimony informative	Substitutions, no.		Results of positive selection test, probability/dN – dS§	Nucleotide diversity, no.
					S	N		
*adk*	387	1	0	0/0	NA	NA	NA	NA
*argA*	357	1	0	0/0	NA	NA	NA	NA
*aroA*	354	1	0	0/0	NA	NA	NA	NA
*glnA*	336	3	0	2/0	10	2	1.000/–2.734	0.01974
*thrA*	339	3	0	2/0	2	0	1.000/–1.455	0.00390
*tmk*	372	4	0	7/0	7	0	1.000/–2.832	0.00941
*trpE*	465	2	0	1/0	1	0	1.000/–1.021	0.00215
Concatemers MLST¶	2,610	6	0	22/5	20	2	1.000/–3.937	0.00289
*inv*	603	2	0	3/0	1	2	1.000/–0.494	0.00498
*yopE*	540	3	0	3/0	0	3	**0.034/1.835**	0.00368
*yadA*	651	3	6	9/2	2	7	**0.408/0.238**	0.00930
Concatemers virulence genes#	1,794	3	6	15/4	3	12	**0.109/1.241**	0.00457
Concatemers MVLST**	4,404	6	6	37/9	23	14	1.000/–3.023	0.00311

*Positive values are shown in bold. dN, number of nonsynonymous substitutions per site; dS, number of synonymous substitutions per site; MLST, multilocus sequence typing; MVLST, multivirulence locus sequence typing; NA, not applicable.  †Housekeeping genes are included in the MLST scheme.  ‡Length of fragments included in the sequence analysis. §The probability of rejecting the null hypothesis of strict neutrality (dN = dS) in favor of the alternative hypothesis (dN is >dS) and the test statistics (dN – dS) are shown. Values were calculated by using MEGA6 *(**10**)*.  ¶Concatemers of sequences included in the MLST scheme. The sequences were cut off to start and finish an analysis with the first and third codon positions, respectively. Concatemers were gathered manually. #Concatemers of virulence gene fragments. **Concatemers of 7 housekeeping and 3 virulence gene fragments.

**Figure 1:**
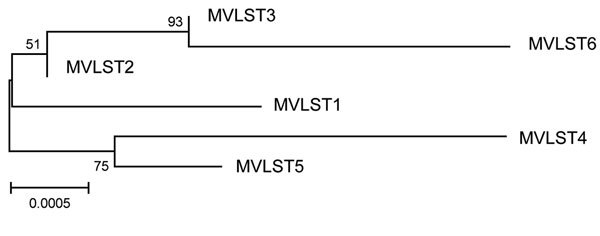
Maximum-likelihood tree generated with concatenated multivirulence locus sequence type (MVLST) sequences for study of Far East scarlet-like fever caused by a clonal group of *Yersinia pseudotuberculosis*, Russia. Reliability values for the branching nodes are indicated. Branch lengths and scale bar indicate distances measured in terms of the proportion of nucleotide substitutions between sequences.

Combining MLST and VST gave rise to 6 multivirulence locus sequence types (MVLSTs) (Table 1). The sequences of 10 MVLST genes (excluding *cnf*) were used to build a maximum-likelihood tree with MEGA6 (*10*). We excluded the *cnf* gene from the analysis because the dominant allele carries a nonsense mutation that interrupts the polypeptide after Asn181. The maximum-likelihood tree divided into 2 subclades (Figure 1). One subclade united MVLSTs found in FESLF isolates and MVLST6, which was found only in rodent isolates. The second subclade united MVLSTs found in rodent and vegetable isolates.

The diversity of virulence genes was analyzed with DnaSP software version 5.10 (*11*; Table 2). A noticeable feature of virulence genes was the predominance of nonsynonymous substitutions, whereas basic parameters of nucleotide diversity were similar in virulence and housekeeping genes (Table 2). Positive selection was confirmed for *yopE* by the Tajima neutrality test implemented in MEGA6. The diversity was especially low among strains from the FESLF subcluster. MVLST1 and MVLST2 shared the VST1 type (Table 1). MVLST3 shared VST2 with MVLST6 found in rodent strains.

Plasmids, particularly the pYV plasmid, are central to the virulence of *Yersiniae* (*12*). The pYV-specific markers *yopE* and *yadA* were found in all strains. The presence of the additional plasmid pVM82/pYpsIP31758.1 was screened with PCR specific to the *dotA* gene (*3*), which was found in all but 4 MVLST1 strains but not in other genotypes. Plasmid purification (*13*) confirmed results obtained from PCR-based screening (data not shown). An additional small plasmid was found in MVLST1 strains that lacked pVM82/pYpsIP31758.1. Consequently, plasmid profiling divided MVLST1 into 2 subtypes, MVLST1a and MVLST1b, without changing other MVLSTs (Table 1).

Our findings show that FESLF clinical manifestations are caused by strains belonging to at least 4 distinct genotypes, with predominance of MVLST1a (MLST2/VST1/pVM82). We consider the MVLST1a genotype to be generally dominant among strains responsible for FESLF in Russia, a suggestion supported by the finding that MVLST1a appears to be the only genotype that carries the pVM82/pYpsIP31758.1 plasmid. A body of epidemiologic data has shown that most epidemic and many sporadic FESLF strains carry this plasmid (*3**,**13*).

The fact that full FESLF symptomatology is caused by several distinct genotypes supports the view that specific virulence traits are characteristic of FESLF-associated strains (*2**,**3*) and suggests that the dominance of the MVLST1a genotype could be caused by its epidemiologic advantages rather than its pathogenic traits. The prevalence of MVLST1a among all isolate sources suggests the genotype’s wider dissemination in the region we studied, which supports the possibility that this clone has epidemiologic advantages. 

To further address this question, we used an evolutionary analysis implemented in MEGA6 (*10*) to test the hypothesis of equality of evolutionary rates by using the χ^2^ test for pairwise comparison of concatenated sequences of MVLST markers, with the *Y. pestis* sequence used as an outgroup. The hypothesis of equal rates between MVLST1 and other genotypes was rejected (p<0.05; Figure 2). The molecular clock test performed with MEGA6 by comparing the maximum-likelihood values with and without molecular clock constraints under the Tamura-Nei model supported this conclusion. The inequality of evolutionary rates favors the idea of more effective reproduction and growth of MVLST1 strains in the environment, possibly because of better adaptation to environmental niches. Another clone with divergent evolutionary rates was the rare MVLST6 (MLST64/VST2) genotype, which has been isolated from small rodents in the Far East of Russia (i.e., in this study and according to data on the isolation of MlST64, listed in the *Y.* pseudotuberculosis MLST database). 

## Conclusions

FESLF, a relatively new disease, is caused by the bacterium that evolved into the causative agent of plague (*14*). The evolution of *Y. pestis* is linked to loss of functionality of some factors that are active in *Y. pseudotuberculosis* and to the acquisition of additional factors of both plasmid and chromosomal origin; these alterations enable the organism to adapt and occupy new environmental niches (*14*). The FESLF causative agent lost at least 2 chromosomally encoded virulence loci, *cnf* and HPI; its most successful clone, MVLST1a, acquired an additional plasmid. The geographic region where the first outbreaks of FESLF were registered seems close, if not identical, to the region where *Y. pestis* emerged. Overall, our data support the view of *Y. pseudotuberculosis* as a rapidly developing pathogenic species, whereas its wide dissemination in the environment promotes selection of clones that are potentially hazardous for humans (*2**–**4**,**15*).

**Technical Appendix.** Additional details of study of Far East scarlet-like fever caused by a few related genotypes of *Yersinia pseudotuberculosis*, Russia.

## References

[1] Grunin II, Somov GP, Zalmover IIu. Far Eastern scarlatinoid fever [in Russian]. Voen Med Zh. 1960;8:62–6. 13709268

[2] Fukushima H, Matsuda Y, Seki R, Tsubokura M, Takeda N, Shubin FN, Geographical heterogeneity between far eastern and western countries in prevalence of the virulence plasmid, the superantigen *Yersinia pseudotuberculosis*-derived mitogen, and the high-pathogenicity island among *Yersinia pseudotuberculosis* strains. J Clin Microbiol. 2001;39:3541–7. 10.1128/JCM.39.10.3541-3547.2001 11574570 PMC88386

[3] Eppinger M, Rosovitz MJ, Fricke WF, Rasko DA, Kokorina G, Fayolle C, The complete genome sequence of *Yersinia pseudotuberculosis* IP31758, the causative agent of Far East scarlet-like fever. PLoS Genet. 2007;3:e142. 10.1371/journal.pgen.0030142 17784789 PMC1959361

[4] Somov G. Far-East scarlet-like fever. Moscow: Medicine; 1979.

[5] Laukkanen-Ninios R, Didelot X, Jolley KA, Morelli G, Sangal V, Kristo P, Population structure of the *Yersinia pseudotuberculosis* complex according to multilocus sequence typing. Environ Microbiol. 2011;13:3114–27. 10.1111/j.1462-2920.2011.02588.x 21951486 PMC3988354

[6] Eitel J, Dersch P. The YadA protein of *Yersinia pseudotuberculosis* mediates high-efficiency uptake into human cells under environmental conditions in which invasin is repressed. Infect Immun. 2002;70:4880–91. 10.1128/IAI.70.9.4880-4891.2002 12183532 PMC128239

[7] Isberg RR, Voorhis DL, Falkow S. Identification of invasin: a protein that allows enteric bacteria to penetrate cultured mammalian cells. Cell. 1987;50:769–78. 10.1016/0092-8674(87)90335-7 3304658

[8] Hoffmann C, Pop M, Leemhuis J, Schirmer J, Aktories K, Schmidt G. The *Yersinia pseudotuberculosis* cytotoxic necrotizing factor (CNFY) selectively activates RhoA. J Biol Chem. 2004;279:16026–32. 10.1074/jbc.M313556200 14761941

[9] Wang X, Parashar K, Sitaram A, Bliska JB. The GAP activity of type III effector YopE triggers killing of *Yersinia* in macrophages. PLoS Pathog. 2014;10:e1004346. 10.1371/journal.ppat.1004346 25165815 PMC4148447

[10] Tamura K, Stecher G, Peterson D, Filipski A, Kumar S. MEGA6: molecular evolutionary genetics analysis version 6.0. Mol Biol Evol. 2013;30:2725–9. 10.1093/molbev/mst197 24132122 PMC3840312

[11] Librado P, Rozas J. DnaSP v5: a software for comprehensive analysis of DNA polymorphism data. Bioinformatics. 2009;25:1451–2. 10.1093/bioinformatics/btp187 19346325

[12] Portnoy DA, Martinez RJ. Role of a plasmid in the pathogenicity of *Yersinia* species. Curr Top Microbiol Immunol. 1985;118:29–51. 10.1007/978-3-642-70586-1_3 3902382

[13] Shubin FN, Gintsburg AL, Kitaev VM, Ianishevskiĭ NV, Zenkova ZG. Analysis of the plasmid composition of *Yersinia pseudotuberculosis* strains and its use for typing pseudotuberculosis pathogens. Mol Gen Mikrobiol Virusol. 1989;6:20–5. 2682219

[14] Achtman M, Zurth K, Morelli G, Torrea G, Guiyoule A, Carniel E. *Yersinia pestis*, the cause of plague, is a recently emerged clone of *Yersinia pseudotuberculosis.* Proc Natl Acad Sci U S A. 1999;96:14043–8. 10.1073/pnas.96.24.14043 10570195 PMC24187

[15] Wren BW. The *Yersiniae—*a model genus to study the rapid evolution of bacterial pathogens. Nat Rev Microbiol. 2003;1:55–64. 10.1038/nrmicro730 15040180

