# Supplementary material for: Far East Scarlet-Like Fever Caused by a Few Related Genotypes of Yersinia pseudotuberculosis, Russia
Source: Emerg Infect Dis. 2016 Mar;22(3):503–6. doi: 10.3201/eid2203.150552 (PMC4766908; doi:10.3201/eid2203.150552)
Supplement: Supplementary file 1 — Technical Appendix. Additional details of study of Far East scarlet-like fever caused by a few related genotypes of Yersinia pseudotuberculosis, Russia. [file 15-0552-Techapp-s1.pdf]

Article DOI: http://dx.doi.org/10.3201/eid2203.150552

Far East Scarlet-Like Fever Caused by a Few Related Genotypes of *Yersinia pseudotuberculosis*, Russia

Technical Appendix

Technical Appendix Table 1. *Yersinia pseudotuberculosis* strains in the study of Far East scarlet–like fever, Russia*

Isolate ID	Strain†	ST		Location‡	Year	Serotype
		MLST	MVST			
FESLF isolates from patients						
1	Ph740 (A)	2	1	Primorye Territory	1973	O1b
2	Ph793 (A)	2	1	Primorye Territory	1973	O1b
3	Ph749 (A)	2	1	Primorye Territory	1973	O1b
4	AH1902	2	1	Anadyr	1973	O1b
5	AH1374	2	1	Anadyr	1973	O1b
6	AH1844	2	1	Anadyr	1973	O1b
7	Spbh823	2	1	St. Petersburg	1975	O1b
8	Spbh817	2	1	St. Petersburg	1975	O1b
9	Kh870	2	1	Kemerovo Region, Angero-Sudzhensk	1975	O1b
10	Kh52 (B)	2	1	Kemerovo Region, Mariinsk	1975	O1b
11	Spbh1373	2	1	St. Petersburg	1975	O1b
12	Kh47 (B)	2	1	Kemerovo Region, Mariinsk	1975	O1b
13	Ph 2204	2	1	Primorye Territory	1976	O1b
14	Ph2128 (C)	2	1	Primorye Territory	1976	O1b
15	Ph2113 (C)	2	1	Primorye Territory	1976	O1b
16	Nh10	2	1	Novosibirsk	1976	O1b
17	Nh1	2	1	Novosibirsk	1976	O1b
18	Yh1	2	1	Republic of Sakha, Yakutia	1977	O1b
19	Yh6	2	1	Republic of Sakha, Yakutia	1977	O1b
20	Yh8	2	1	Republic of Sakha, Yakutia	1977	O1b
21	Yh29	2	1	Republic of Sakha, Yakutia	1977	O1b
22	Sh426	26	2	Sakhalin Island	1977	O1b
23	Sh525	26	2	Sakhalin Island	1977	O1b
24	Sh33	26	2	Sakhalin Island	1977	O1b
25	Ph40D (D)	32	3	Primorye Territory	1980	O3
26	Ph33D (D)	32	3	Primorye Territory	1980	O3
27	Ph3D (D)	32	3	Primorye Territory	1980	O3
28	Ph512	2	1	Primorye Territory	1980	O1b
29	Ph84	2	1	Primorye Territory	1989	O1b
30	Ph64	2	1	Primorye Territory	1989	O1b
31	Ph7530	2	1	Primorye Territory	1994	O1b
32	Ph2053	2	1	Primorye Territory	2008	O1b
33	NH9 (E)	2	1	Novosibirsk	2011	O1b
34	NH37 (E)	2	1	Novosibirsk	2011	O1b
35	NH44 (E)	2	1	Novosibirsk	2011	O1b
36	NH36 (F)	2	1	Novosibirsk	2011	O1b
37	NH39 (F)	2	1	Novosibirsk	2011	O1b
38	NH42 (F)	2	1	Novosibirsk	2011	O1b
39	NH46 (F)	2	1	Novosibirsk	2011	O1b
40	ps-1	2	1	Vladivostok	2014	O1b
41	ps-3	26	2	Vladivostok	2014	01b
Isolates from wild rodents						
42	PR1759-60	2	1	Primorye Territory	1987	O1
43	PR92	2	1	Primorye Territory	1987	O1
44	PR177	2	1	Primorye Territory	1988	O1
45	PR152	2	1	Primorye Territory	1988	O1

Isolate ID	Strain†	ST		Location‡	Year	Serotype
		MLST	MVST			
47	PR184	2	1	Primorye Territory	1988	O1
48	PR1694-98	42	4	Primorye Territory	1994	O1
49	PR105-06	42	4	Primorye Territory	1997	O1
50	PR76-78	42	4	Primorye Territory	1998	O1
51	YR1498	2	1	Yakutia	2009	O1
52	YR1497	2	1	Yakutia	2009	O1
53	YR1500	2	1	Yakutia	2009	O1
54	YR1330	2	1	Yakutia	2010	O1
55	PR220	32	1	Primorye Territory	2011	O1
56	PR96-97	64	5	Primorye Territory	2011	O1
57	PR95	64	5	Primorye Territory	2011	O1
Isolates from vegetables						
58	PV252	2	1	Primorye Territory	1977	O1
59	PV270	2	1	Primorye Territory	1977	O1
60	PV238	2	1	Primorye Territory	1977	O1
61	PV249	2	1	Primorye Territory	1977	O1
62	PV673	2	1	Primorye Territory	1978	O1
63	PV608	2	1	Primorye Territory	1978	O1
64	PV75	2	1	Primorye Territory	1978	O1
65	PV162	2	1	Primorye Territory	1978	O1
66	PV82	2	1	Primorye Territory	1980	O1
67	PV696	14	6	Primorye Territory	1981	O1
68	PV177	2	1	Primorye Territory	1988	O1

*FESLF, Far East scarlet-like fever; MLST, multilocus sequence type; MVLST, multivirulence locus sequence type; ST, sequence type.
†Outbreak strains are shown by a letter in parentheses.
‡Technical Appendix Figure 2 shows locations in Russia.

Technical Appendix Table 2. Primers used in the study of Far East scarlet-like fever caused by *Yersinia pseudotuberculosis*, Russia

Gene	Primer sequence, 5′→ 3′
inv	TATGGGGACCCGCAGGCTGGC
	TGCCGCCATCGTATATCCACCG
yadA	CATATGCATTTGCCGAGGAGC
	GAGTGTTTTTTGGCCGCATCC
yopE	ATGCAAACAATCTGGCCGGGC
	CCACTGCGAGAAGGGAATACC
cnf	GCAGGTGGGAGCAACAAAGAT
	CAGGAGCGAACAACTAAATGGAA
dotA	ATTGAGCCTATAACACCCGTA
	ATCGGTTAATACTGTCGCTGA

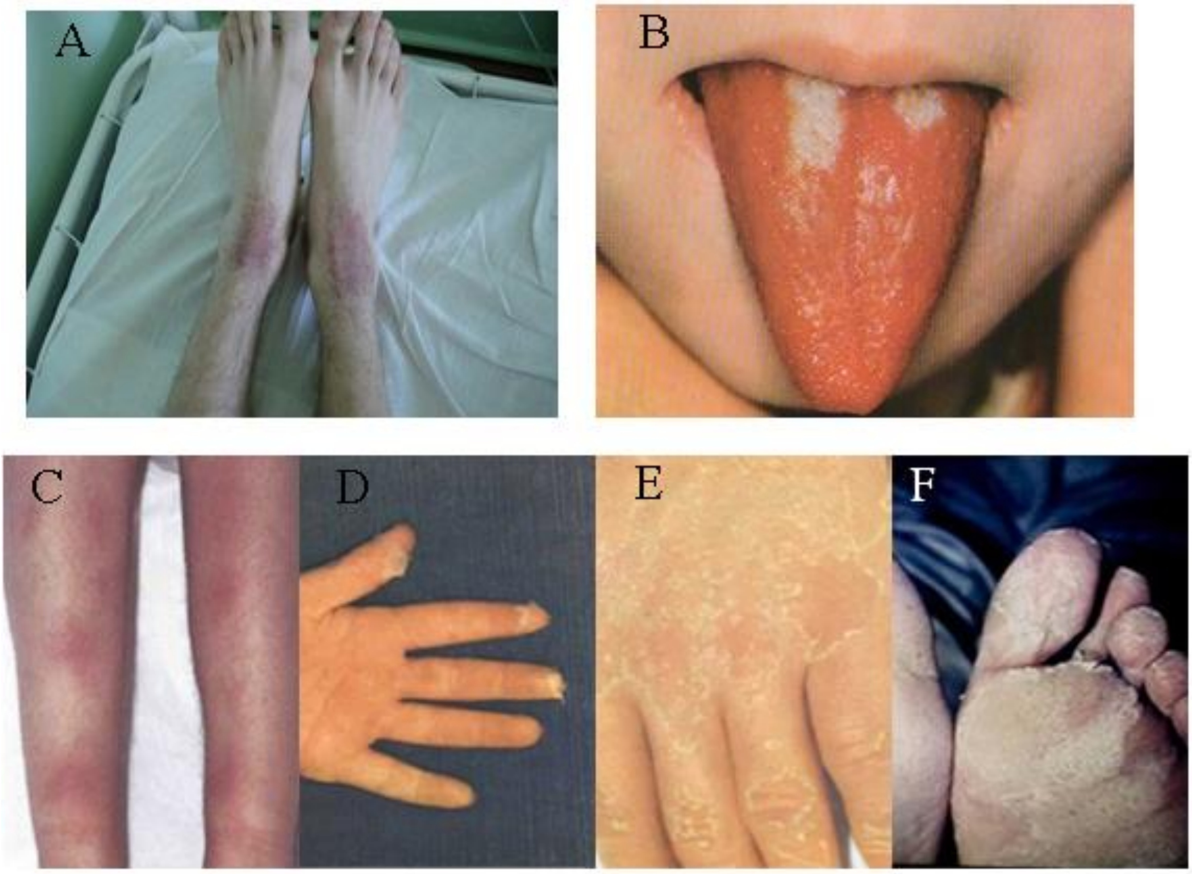

Technical Appendix Figure 1. Visible clinical manifestations of Far East scarlet-like fever. A, B) Early signs and the acute period of the disease: rash and “raspberry tongue”; C) relapse period: erythema nodosum; D–F) recovery period: defurfuration.

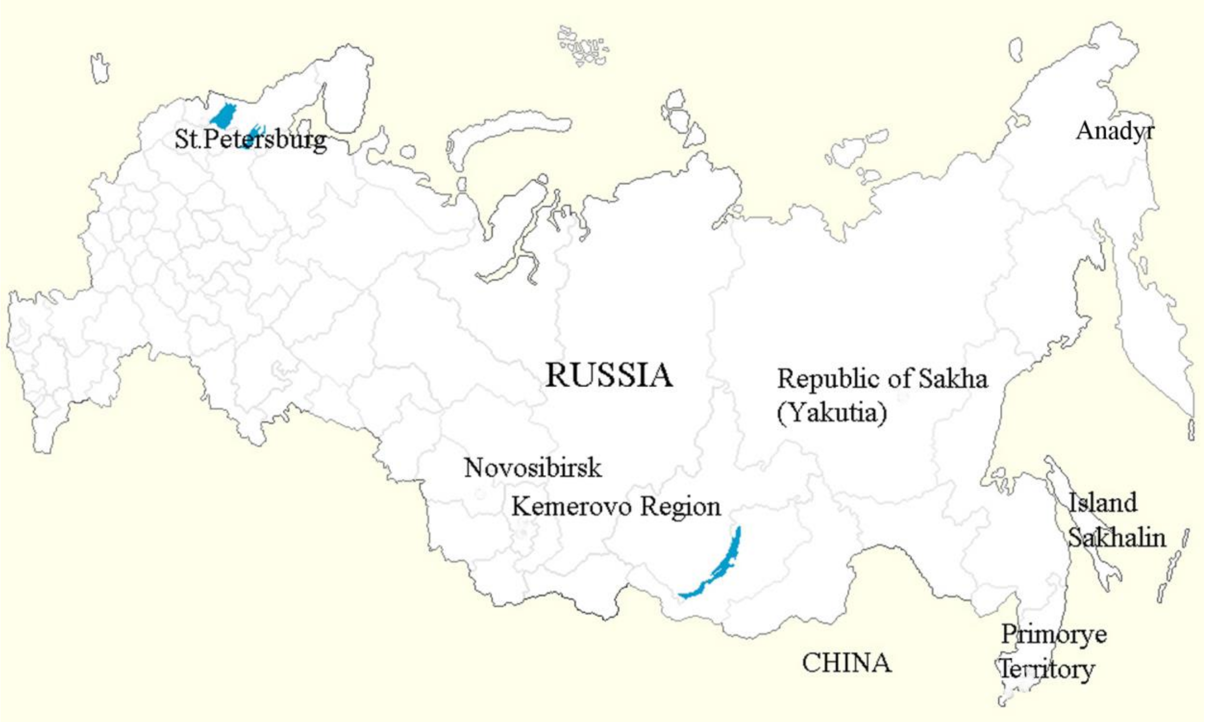

Technical Appendix Figure 2. Map of Russia showing locations of *Yersinia pseudotuberculosis* strains in the study of Far East scarlet-like Fever, Russia (see Technical Appendix Table 1).
